# Mad River Dental Society

**Published:** 1885-07

**Authors:** E. G. Betty


### Proceedings and Discussions.

### Mad River Dental Society.

REPORTED BY E. G. BETTY, D.D.S.

The last annual meeting of this society was called to order by President Paine, at 10 o'clock A.M., May 19th, 1885, in the parlor of the Phillips House, Dayton, O. The reading of the minutes of the previous year's meeting was dispensed with, the Secretary not being present, and on motion of Dr. Watt, Dr. Betty was appointed Secretary *pro tem.* Dr. Taft suggested that a committee of two on membership be appointed; carried, the chair appointing Drs. Elson and J. H. Paine.

Dr. Paine, of Middletown, read part of a paper on "Dental Economics," in which he considered the proprieties of various matters pertaining to daily practice.

*Dr. Taft.* This subject is one of unusual presentation, and I wondered what suggested the subject—upon second thought, it may be profitable to discuss it, at least some parts of it.

Economics is that science which treats of the best use of resources; all branches of human ingenuity and industry have their economics. What are some of the resources of the dentist? Knowledge is one of his resources, and on this he mainly relies to accomplish the duties and work of his profession; how shall he best use this knowledge, take care of it, and increase his store? Economics also implies the improvement of our resources and the increase of them.

If knowledge is one of our resources as dentists, it behooves us to increase, multiply, and improve our stock for the use and benefit of those who call upon us to administer relief. The recent graduate is very apt to think he is at the top of the ladder of professional knowledge, but, as year after year passes over his head, he finds that it is necessary for him to continually augment his stock of information or he will be left behind by those who are pushing forward. We often encounter obstacles which prevent us from applying our knowledge at its best; prominent among these is ill health, this unfits us for the best exercise of our powers and it devolves upon us to maintain our physical condition at the best, so that we can obtain the highest results from the application of our knowledge.

If we assume to treat disease, we should have our knowledge in the best condition for use in order to secure the best result, and to that end we must continue to increase and improve our store, constantly adding to it new facts and new materials, and make them available as well. Knowledge is within the reach of all. Our literature, both periodical and textual, is within the reach of every one, and from it we should glean all that is new, valuable, and of practical import. We need not only *new* things, but a thorough knowledge of old ones as well, because it makes us better judges and more capable of analyzing new ones. The things we glean from our literature, we assimilate and appropriate; they then become a part of ourselves, cropping out in our work and all that we undertake, in that way being productive of the greatest possible good. If knowledge is one of our resources, so are appliances, instruments, and agents amployed for treating disease both in its therapeutical and surgical aspect. In our selection of that agent or instrument to use, we should bring to bear a knowledge of *principles*, looking toward the accomplishment of the end in view. But we must not expect, much less try to "get off easy," for we must do that which is most reliable and capable of producing the *best* results in the case. *This* is true economy, it may not be the best for us financially just now, but in the future it will return a suitable reward and one not to be measured by dollars and cents.

Let us have the best and most perfect instruments and appliances we can obtain. Would we in the majority of the offices throughout the country find the *best* appliances? I fear not, not even in a reasonable proportion of the offices. In very many things we recognize the principle that, "the best is the cheapest," so, too, in the accomplishment of the results we so much desire.

There is a broad field for thought and study, investigation and research in therapeutics, remedial agents, medicaments, etc. The rage for new things has a tendency to and does crowd out the old ones that are oftentimes superior to the new. In this matter, we should be careful to discriminate and not be too hasty in the adoption of a new thing without an adequate knowledge of its uses and application, as we often fail for want of the knowledge that would render the agent efficient and reliable, consequently becoming discouraged and abandoning many things that are really of value if properly understood and applied.

Our knowledge, our appliances, our life, our health, *all* should be used to the advantage of our profession, we will then achieve the highest success.

*Dr. Watt.* Dr. Berry's remark about the uselessness of a thermometer on his vulcanizer reminds me of * * * who when told by a friend that in raising steam in his boiler, for safetys' sake, enjoined his fireman (a lazy one) not to fire up *too* much, and to use green wood, replied, "put on a steam gauge."

*Dr. Murray.* I would like to ask Dr. Paine if he regards it as unprofessional to play baseball, groom a horse, or take a ride?

*Dr. J. H. Paine.* I do not wish to make too many hard remarks about the young men, but I do *not* think it is proper to play baseball; as regards the horse, if you *own* him, you may act the groom.

*Dr. Taft.* A few remarks about the system and order that should be observed in a dental office. Perfect order and system facilitate operations and save time, all instruments should be of the very best make and quality, and so arranged as to be easily reached; they should be classified according to kind and each one have a distinct place, and be always in it when not in use. Furthermore, the operator should be so thoroughly familiar with them and their places that he can find any given instrument in the dark.

There should not be a surplus of instruments in the case, useless ones, those worn out or broken. The number should be the smallest compatible with the purpose to be served. All instruments occupying room in the case should always be kept in firstclass working order, the chisels and excavators sharp, spatulas clean, etc.; all of which tend to economize time, labor, and temper.

It is of supreme importance to preserve health and strength. Let each one select and follow that exercise or amusement which is most congenial to his inclination and best suited to his bodily strength, one with horse-back riding, another with bees, another with fishing, and so on.

*Dr. C. H. James.* We should all select that exercise which is best suited to our needs and physique, it is much better to have a moderate, regular exercise than that which is intense and only occasional.

I was very much pleased with Dr. Berry's little paper, it sounded very much like old-fashioned office-talk, and more especially was I pleased with his recommendation of the "Buckeye" vulcanizer, as I had the honor to invent that machine, though I have never reaped any pecuniary benefit from it. I am still using one that I had made twenty years ago, one of the very first, and it is still in good working order.

While I am on my feet I should like to invite every one here to go to Chillicothe next October and be present at the perfecting of the reorganization of the Ohio State Dental Society. We are going to make a "go" of it this time sure and every one ought to feel a pride in the State Society and be present to take part for he will not regret it.

*Dr. Paine.* Another point in economics is the saving of time, this can be done by appointments, as I sometimes have to work for some one to whom I am indebted when a cash patient comes in, and I greatly prefer the cash because the "dead-head" will come back of his own accord.

*Dr. Wilson.* System is everything, both in our offices and in our studies.

*Dr. James.* The medical profession and the public are better acquainted with their mutual relations than the dentists and the public, and the time has come when the dental profession and the public should have an adequate knowledge of what they are to expect from each other.

Adjourned till 2 P.M.

AFTERNOON SESSION.

At the appointed time the members assembled, and the President being absent, Dr. J. Taft was elected President *pro tem;* the minutes of the previous year's meeting were read and approved, as also were those of the morning session.

Dr. Watt then read a paper on "No New Thing under the Sun," from proof sheet.

On motion of Dr. Sillito it was decided to reduce the annual dues to fifty cents.

The third subject was announced, "Different Methods of and Different Materials for Filling Teeth," upon which Dr. Betty read a paper.

*Dr. Berry.* Oxy-phosphate can be hardened very nicely with the warm air current.

*Dr. Wilson.* In the first class of cases mentioned in the paper, instead of filling *all* the cavity with the oxyphosphate, I cover the cervical border with Hill's stopping as it does not wash away.

*Dr. Murray.* I would like to ask Dr. Watt if there is any difference in the solubility of oxy-phosphate and Hill's stopping?

*Dr. Watt.* Hill's stopping is largely composed of gutta-percha, which substance is not soluble in the saliva.

*Dr. Berry.* Hill's stopping, in my opinion, is preferable to any of the preparations of oxy-phosphate, as the hardening process that goes on under it is remarkable, and, furthermore, it does not receive the credit from the profession that its merits deserve.

*Dr. Watt.* I have dissolved gutta-percha in chloroform, mixed in bone phosphate and filled temporarily with the preparation. I think that neither tin nor gutta-percha have received the amount of attention they deserve. In children's teeth, where the walls are friable and the cavity exposed to grinding, it will be much better to fill with tin.

*Dr. Paine.* In 1858 I filled a tooth for a girl with tin, and last week upon examining it, I found that both tooth and filling are in very good condition.

*Dr. Bradley.* Oxy-phosphate alone should not be discussed, as there are other materials that require our consideration. The question is, "with what material are we doing the most good?"

*Dr. J. Taft.* The *manner* in which the oxy-phosphates are used, or should be used, is worthy of note. Probably the best method of making the mixture is to put out the necessary amount of the powder upon a slab of glass or porcelain and place the liquid beside it, then mix the two together by thorough rubbing with a broad spatula. This will produce a thorough incorporation of the ingredients, which will then make a most smooth, solid, and compact filling and one capable of withstanding three or four year's wear, outlasting others that have been but indifferently prepared and in the mouth but a year. In introducing the mass into the cavity it should be carefully manipulated and well adjusted into its place, and not hastily or carelessly jammed into the cavity as is frequently done. Cavities carefully filled in the manner I have suggested will show little or no more wear at the cervical border after a considerable period, than when first introduced. Upon the most thorough manipulation depends the most successful and satisfactory result. In very large cavities in the crowns of molars that many say can only be filled with amalgam, remove all *debris* and diseased tissue. When this is done, take a bit of gold (pure) plate, about thirty of Stubb's gauge and fit it to the orifice of the cavity, burnishing over the edges to make it fit snugly, then solder on two or three little loops for anchors, or old platinum pins will answer the purpose very nicely—the cavity is then filled with oxy-phosphate flush with the top or borders, the gold cap then pressed down to its place firmly and evenly, allowing time for the escape of the surplus cement which can be trimmed away when it has thorougly set; the edges of the cap may now be accurately fitted by burnishing down with a suitable instrument. An operation performed in this way serves all the purposes of a gold filling made over oxy-phosphate, is inexpensive to the patient, and does not consume the amount of time required to introduce foil; in my own mouth I would prefer it to a filling of equal size made of solid gold.

*Dr. Runyan.* It is at the cervical border that oxy-phosphate gives way, and in order to prevent that I adjust a cap at that border and fill over it with the cement. [What kind of a cap was not stated.—Rep.]

*Dr. Taft.* If you will manipulate the oxy-phosphate in the manner I have described, you will not experience the trouble at the point you mention. I have seen caps of Hill's stopping at the cervical border look as soft as though they had been soaked in chloroform.

*Dr. Berry.* Dr. Taft's remarks about the mixing and manipulation of oxy-phosphate have, it seems to me, about hit the thing fair and square. As for myself, I mix the preparation somewhat thin at first, rubbing the powder into the liquid very thoroughly and for a long time, and when it has reached the proper consistency for introduction into the cavity, I adapt it carefully and snugly to the walls and borders; this method I think makes these fillings more durable, especially if the filling is kept dry as long as possible after being put in.

*Dr. Adams.* In these large cavities I always put about one-third oxyphosphate, then a layer of Robinson's fibrous foil over that, covering the top with gold.

*Dr. Taft.* I don't see any especial advantage in making a filling of three different materials as Dr. Adams describes, it is sufficient to carry the phosphate up higher and then cover with gold.

*Dr. N. S Hoff.* In my opinion, we often make mistakes in the preparation of cavities to be filled with plastics of all kinds, and I have lately adopted a new method of shaping cavities, especially those I intend to fill with amalgam. All approximal cavities that are very difficult to keep dry, I fill with the aid of a matrix made from a round copper wire, hammered on the horn of an anvil.* This matrix is of peculiar shape, and can be very nicely and closely adapted to the borders, and can easily be high up between the gum and the tooth, thus securing that dryness which is of such prime importance to all fillings, oxy-phosphate no less than others. A filling of this material may be left

*This matrix was described in "Salmagundi" for January, 1884.

as it comes from the instrument with which it is introduced, but I prefer to dress down carefully and do not think that it detracts any from the durability of the phosphate to do so.

Some time ago I read an article in a dental journal, an Eastern one I think, elaborating the theory that amalgam fillings fail from a tendency the amalgam has to assume the spheroidal or globular shape that mercury itself does. The idea struck me as somewhat peculiar and not without foundation, and to counteract this tendency as much as possible I prepare the cavities as round as I can. Instead of cutting away freely from the grinding surface of an approximal cavity in a molar or bicuspid as I do for gold, I only remove enough of that surface to give the necessary room to introduce the excavator and the cavity bur. The lateral and cervical walls are trimmed in such a way as to make the orifice of the cavity round, contracting as it approaches the grinding surface, and this being more or less pear-shaped. The anchorages for the filling (under cuts), I make with a round burr, thus making the interior as like a hollow sphere as possible. All this I do in conformity with the theory I mention; there may be something in it though I have not pursued it long enough to make any positive statements from experience.

*Dr. Berry.* I often have occasion to use a matrix and make one of a lamina of mica, pressing it into place, under the gum if necessary, and then introducing the filling. I like it very much.

*Dr. C. H. James.* Dr. Taft's method of covering with a gold cap is very good, but we have now become so dextrous in the manipulation of gold foil that we might as well in these cases, fill nearly full with oxy-phosphate and then build on the top with gold foil.

*Dr. Taft.* The method Dr. James mentions takes more time and costs the patient more money than the one I described. Dr. Adams said something about his method of filling a little while ago, but I do not think there is any advantage in it; better use the oxyphosphate alone or cap with foil. I have used Robinson's foil torn up into shreds and mixed with oxy-phosphate, and I think it is better than oxy-phosphate alone. The phosphate should *never* be dressed down, but left just as it is introduced.

*Dr. Betty.* It seems to have been forgotten that the phosphate may be trimmed as much as the operator may desire or the case require, and then polished with a piece of heated talc (soapstone), this making a good, hard, and durable surface.

*Dr. Taft.* That is all very true, but the filling can be made just as dense by rubbing with the instrument with which it has been introduced.

*Dr. Watt.* I want to say something about amalgam too, you you all know I *love* amalgam. The colors that are produced around an amalgam filling in the mouth are the result of chemical action by sulphur, oxygen, or chlorine. The sub-oxide of mercury is black as also is the oxide of silver, so this cannot be caused by copper; if you examine the color you can tell the metal producing it.

*Dr. Wilson.* Dr. Taft may mix oxy-phosphate so as to make it last as long as amalgam, but I find that it does not work equally as well in all cases, as mouths differ. As to amalgam, I prepare the cavity *for* amalgam, mixing and using it dry. There is one class of cases in which amalgam can be used very successfully, and that is, where decay has taken place under a gold filling. The amalgam will turn very black in all such instances. The temporary teeth I fill with oxy-phosphate, Hill's stopping, or gutta percha. While I am on my feet I would like to ask if it is good practice to cut away good sound tooth substance just for the sake of putting on a cap of gold, especially when no elongation of the tooth is intended?

*Dr. Taft.* If the process of wasting away is arrested, the practice is good, but if the whole end of the tooth is not protected and the wasting away continues, then the practice is of little account. If it is desired to protect the whole end of the tooth and yet not produce any elongation, it can be done by grinding the edges of the enamel sufficiently to make room for the necessary amount of gold to protect the tooth. The conservative treatment of the temporary teeth is important, and nearly all know that it is necessary to protect them until the natural process of shedding takes place. If the time of the eruption of the second teeth is not far off, then either oxy-phosphate or

Hill's stopping will answer the purpose, but if it is somewhat distant, it is better to fill with tin. The teeth should be examined frequently so that anything that goes wrong may be corrected before serious damage results.

*Dr. Sillito.* Is it necessary to fill out the fissures from the central cavity?

*Dr. Taft.* Yes, if permanent work is to be done; cut them out whether decayed or not, and fill them with tin; this applies to the permanent molars in mouths of children.

*Dr. Wilson.* I sometimes use gold and platinum foil in building caps upon abraded incisors, but I object to the gray color, as patients are apt to think the filling is not of gold.

The subject was passed and the Society proceeded to elect officers for the ensuing year with the following result:

President, C. Bradley, Dayton, Ohio.
Vice-President, F. C. Runyan, Springfield, Ohio.
Secretary and Treasurer, L. C. Adams, Dayton, Ohio.

Drs. N. S. Hoff and E. G. Betty were appointed an auditing committee and found the accounts and funds correct.

*Dr. Murray.* I would like to hear from some of the gentlemen present as to what has been their experience with muriate of cocaine. I used a four percent solution in one case and was enabled to remove part of a pulp from a root without pain.

*Dr. Berry.* Dr. Wright performed an operation successfully, the remedy being topically applied.

*Dr. Watt.* There is not a doubt but that there is merit in muriate of cocaine, but you must have a reliable article as it is easily decomposed and of course loses its potency.

The extraction of a tooth, while it lasts, is one of the most painful operations of surgery and it has to be done occasionally (often more so), so that it is right and proper that the pain should be lessened. I formerly used electricity to obtund the pain, and out of 2,500 recorded cases, I found in 500 no diminution of pain; in as many others or more, the shock itself produced pain and rendered the extraction itself more painful. [The reporter did not catch the result in the balance of the cases.]

The contrary results obtained from the use of cocaine are due to the fact of its being so easily decomposed, and the consequent unreliability of the article.

*Dr. Berry.* I used to use electricity in the extraction of teeth, but with indifferent results.

*Dr. Bradley.* I have had remarkable results from the administration of English stock ale as it seems to produce a stupefaction and indifferance to pain. I have used whisky and brandy, but would not advise any one to use them.

*Dr. Watt.* I suppose it was the lupulin contained in the hops from which the ale is made that produced the stupefaction.

*Dr. Berry.* I used brandy in one case and the patient thought it was chloroform, because she had asked for it.

During the course of the meeting the following gentlemen were elected to membership: Drs. C. A. Murray, Delphos O.; Homer Ainsworth, Van Wert, O.; C. C. Scott, Celina, O.; A. R. Cuyler, Dayton, O.; N. S. Hoff, Cincinnati, O.; W. E. Tizzard, Dayton, O.; C. H. James, Cincinnati, O.; W. H. Creighton, Jamestown, O.; W. N. Wilson, Richmond, Ind.

Adjourned to meet again on the third Tuesday in May, 1886, at Dayton, O.

